# Influence of Reproductive Status on Tissue Composition and Biomechanical Properties of Ovine Vagina

**DOI:** 10.1371/journal.pone.0093172

**Published:** 2014-04-07

**Authors:** Daniela Ulrich, Sharon L. Edwards, Kai Su, Jacinta F. White, John A. M. Ramshaw, Graham Jenkin, Jan Deprest, Anna Rosamilia, Jerome A. Werkmeister, Caroline E. Gargett

**Affiliations:** 1 The Ritchie Centre, MIMR-PHI Institute of Medical Research, Melbourne, VIC, Australia; 2 Department of Obstetrics and Gynecology, Monash University, Melbourne, Victoria, Australia; 3 CSIRO Materials Science Engineering, Melbourne, VIC, Australia; 4 Department of Obstetrics and Gynecology, University Hospitals Leuven, Leuven, Belgium; UT-Southwestern Med Ctr, United States of America

## Abstract

**Objective:**

To undertake a comprehensive analysis of the biochemical tissue composition and passive biomechanical properties of ovine vagina and relate this to the histo-architecture at different reproductive stages as part of the establishment of a large preclinical animal model for evaluating regenerative medicine approaches for surgical treatment of pelvic organ prolapse.

**Methods:**

Vaginal tissue was collected from virgin (n = 3), parous (n = 6) and pregnant sheep (n = 6; mean gestation; 132 d; term = 145 d). Tissue histology was analyzed using H+E and Masson's Trichrome staining. Biochemical analysis of the extracellular matrix proteins used a hydroxyproline assay to quantify total collagen, SDS PAGE to measure collagen III/I+III ratios, dimethylmethylene blue to quantify glycosaminoglycans and amino acid analysis to quantify elastin. Uniaxial tensiometry was used to determine the Young's modulus, maximum stress and strain, and permanent strain following cyclic loading.

**Results:**

Vaginal tissue of virgin sheep had the lowest total collagen content and permanent strain. Parous tissue had the highest total collagen and lowest elastin content with concomitant high maximum stress. In contrast, pregnant sheep had the highest elastin and lowest collagen contents, and thickest smooth muscle layer, which was associated with low maximum stress and poor dimensional recovery following repetitive loading.

**Conclusion:**

Pregnant ovine vagina was the most extensible, but the weakest tissue, whereas parous and virgin tissues were strong and elastic. Pregnancy had the greatest impact on tissue composition and biomechanical properties, compatible with significant tissue remodeling as demonstrated in other species. Biochemical changes in tissue protein composition coincide with these altered biomechanical properties.

## Introduction

Pelvic organ prolapse (POP) is a common condition affecting millions of women worldwide. Up to 35% of all women in the US have one or more symptoms of POP frequently involving urinary incontinence [1]. POP is defined as the herniation of the bladder, the uterus or the bowel into the lower genital tract [2] caused by several known and unknown factors. The etiology is not fully understood, but vaginal childbirth trauma is a known risk factor; hormonal status may also be relevant [3]. The support structures of the pelvic floor include the pelvic floor muscles, the cardinal and uterosacral suspensory ligaments and the dense fibromuscular connective tissue of the vaginal wall [4]. The cellular components of the vaginal wall comprise fibroblasts which produce the majority of the extracellular matrix (ECM), and smooth muscle cells which function in vaginal contractility. The ECM consists of fibrous proteins (e.g. collagens, elastin), as well as a range of glycosaminoglycans (GAGs) [5]. The ECM is dynamic and responds to changes in the environment by remodeling. Collagen type I comprises the major fibrillar protein found in bone, ligaments and interstitial tissues and contributes to their tensile strength [6]. Collagen I, together with elastin and smooth muscle cells influence the biomechanical and particularly the viscoelastic properties of the vaginal wall. Collagen type III is also a fibrillar protein widely distributed in soft tissues and can contribute to tissue elasticity. An increase in collagen III in healing or regenerating tissues usually reduces its mechanical strength by decreasing the overall collagen fiber diameter of collagen I/III heterofibrils [7].

Studies of the human vaginal wall especially in the non-prolapsed state have been limited by lack of information on the exact site of tissue acquisition and are often restricted to one type of analysis. Sheep have been suggested as a convenient model for assessing surgical treatments for POP [8]. The advantage of the ovine vaginal model is that the pelvic tissue anatomy is similar in size and structure to humans. Sheep have prolonged labors with relatively large fetuses and spontaneously develop prolapse related to vaginal birth [8], [9]. Similar to humans, sheep develop POP over a wide range of ages, but predominantly in the months following delivery. The incidence is greatest in multiparous sheep and increases with parity [8]. However the model is still poorly defined despite these similarities between sheep and humans.

The aim of this study was to undertake a comprehensive analysis of the biochemical tissue composition and biomechanical properties of ovine vagina and relate this to histo-architecture at different reproductive stages as part of the establishment of a large preclinical animal model for evaluating regenerative medicine approaches for transvaginal surgical treatment of pelvic organ prolapse. A secondary aim was to determine if there were any differences in these parameters between the anterior and posterior vaginal walls, given that bladder involvement occurs in nearly half the cases of ovine POP.

## Materials and Methods

The experimental procedures and sheep husbandry were approved by the Monash Medical Centre Animal Ethics Committee A (MMCA 2011 45). Border Leicester Merino (BLM) sheep were housed in the animal house of Monash Animal Service facilities in compliance with the National Health and Medical Research Council of Australia guidelines for the care and use of laboratory animals. The sheep were housed in barns with unlimited food and water supply.

Vaginal tissue was harvested from 3 groups of BLM sheep, virgin, parous (sheep that delivered at least three singleton lambs vaginally), and parous pregnant (3rd stage of pregnancy in sheep that also had delivered 3 singleton lambs vaginally prior to the current pregnancy). All animals were humanely euthanized according to the current guidelines by intravenous administration with Pentobarbitone sodium into the jugular vein (150 mg/kg).

**Figure 1:**
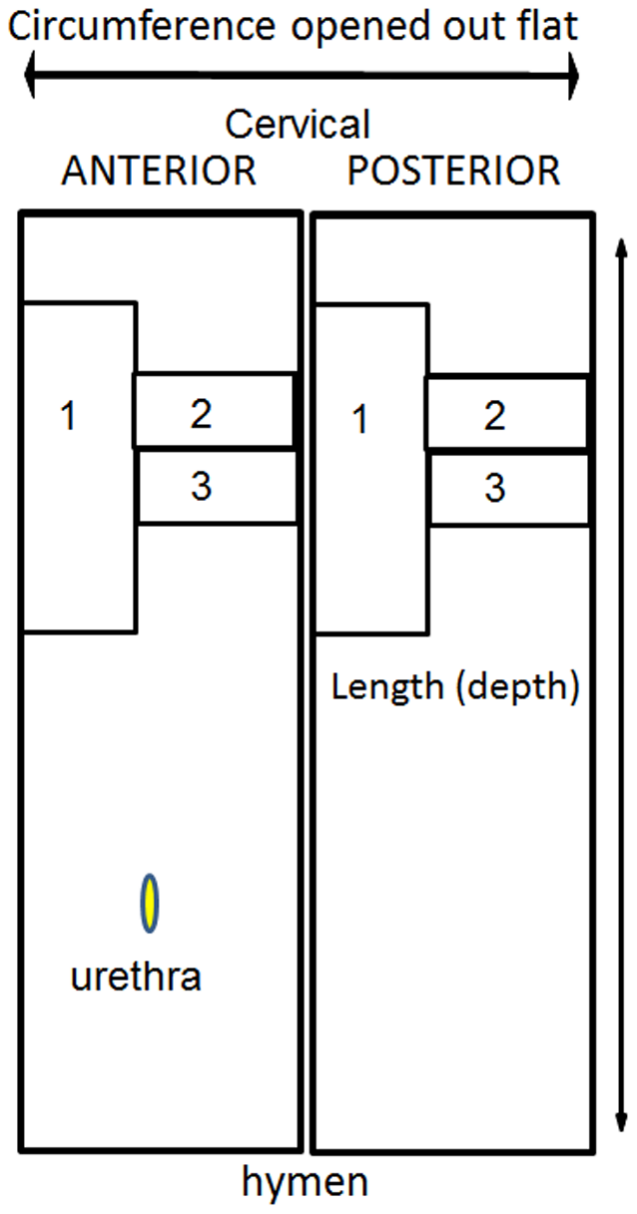
Schematic showing dissection of ovine vaginal tissue for 1 biomechanical testing, 2 histology, 3 biochemical analysis.

A measure of maximum displacement of the vaginal wall was established to quantify vaginal wall distensibility. Ewes were placed in the dorsal recumbency position and values were obtained for points corresponding to Ba, Bp and C of the POP-Q in humans [10] by clamping the tissue and traction applied to the vaginal walls approximately 3 cm above the external urethral meatus and to the cervix. The level of maximum displacement was measured in centimeters with a ruler. The complete vaginal tract was immediately excised and full thickness vaginal tissue was collected in a longitudinal manner from the upper third of the anterior and posterior vaginal wall and divided into 3 parts for biomechanical, biochemical, and histological analysis as shown in Figure 1.

The explanted tissue for histological analysis was fixed in 4% paraformaldehyde (PFA) for 24 hours, then oriented and embedded in paraffin to obtain full vaginal thickness, sectioned into 5 µm sections and stained with hematoxylin and eosin (H+E) and with Masson's Trichrome. For measurements, only images that showed a full depth (complete set of histological components) were analysed. The four zones in the images were readily identified using a Trichrome stain. In all cases measurements were taken on the axis perpendicular to the epithelium. The height of the muscularis was measured using NIS-elements RA3.2 software. The maximum and minimum thickness for the muscularis and vaginal wall (epithelium to adventitia) was measured on a single section for each sample for both anterior and posterior walls, and the mean and SEM calculated for each experimental group. For immunohistochemistry the tissue was fixed, embedded and sectioned as above and stained with alpha smooth muscle actin (αSMA) (Dako, Glostrup, Denmark,) to label smooth muscle cells and myofibroblasts. Sections were dewaxed and rehydrated, and protein block (Dako®) was applied for 30 minutes at RT. After three washes in PBS, sections were incubated with the primary antibody for one hour at 37°C at 1∶400 dilution. Mouse IgG1 isotype (Dako) was used for the negative control and applied at the same concentration. Bound antibodies were detected with secondary Streptavidin HRP-conjugated antibody (AbD Serotec ®, Oxford, UK) for 30 minutes at RT after 3 washes in PBS. 3,3′-Diaminobenzidine (Sigma-Aldrich ®, St. Louis, MO, USA) was used as a chromogen. The slides were dehydrated in graded alcohols and mounted with DPX mounting medium.

The biochemical components were determined in triplicate for each sample for the following assays. Total collagen content was measured by hydroxyproline (Hyp) assay. Freshly excised tissue pieces (5×5 mm) were frozen at −20°C. Tissue pieces were then pre-weighed and lyophilized for 4 hours, wet weight is shown in Figure S1. The dry weight (W_dry_) was recorded and the water content of the original tissue calculated. The dried tissue was digested with papain (0.5 mg/ml in 0.1 M Na2HPO4, 5 mM EDTA, 5 mM L-Cysteine HCl, pH 7.4) for 16 hours. After centrifuging, the supernatants were hydrolyzed in 6N HCl at 115°C for 4 hours and then desiccated overnight. Hyp was measured spectrophotometrically at 560 nm after reaction with 0.05 mol/L chloramine-T (Sigma) and 10% (w/v in 2-methoxyethanol) ρ-dimethylaminobenzaldehyde (Sigma) [11]. A standard curve using L-hydroxyproline (0–10 µg/mL) (Sigma) was used to calculate the Hyp concentration. Total collagen was calculated using a hydroxyproline to collagen ratio of 0.143∶1 [11].

The ratio of collagen type III to collagen type I was determined by an SDS-polyacrylamide gel electrophoresis (SDS-PAGE) using delayed reduction [12] as previously described [13]. Frozen vaginal tissue was thawed to room temperature (RT) for 15 min, and 5×5 mm pieces adjacent to the area excised for biomechanical analysis (Fig. 1) were digested for 4 h in pepsin (Sigma) (0.5 mg/ml in 100 mM acetic acid, pH 2.5) at 4°C followed by brief homogenization with an T10 basic Ultra-Turrax® (IKA®, USA) homogenizer. Samples were further digested in the pepsin solution for 16 h. After centrifugation, 10 µl of each sample was mixed with 40 µl NuPAGE® LDS sample buffer (Life Technologies), heated to 90°C for 1–2 min and then loaded onto NuPAGE 4–12% Bis-tris gels (Life Technologies) with MES running buffer (Life Technologies); 50 mM (2-[N-morpholino] ethane sulfonic acid) in 50 mM Tris base (1 mM EDTA, 0.1% SDS; pH 7.3). Samples were electrophoresed for 1 h at 130 V, the power turned off, and 5% v/v 2-mercaptoethanol (Sigma) was added to each well for 1 h. Finally electrophoresis was continued for 3 h at 130 V at 4°C. Gels were stained with Coomassie Blue R-250 solution destained in 20% ethanol and 5% acetic acid. Images were taken using FujiFilm LAS-3000 software. The percentage of collagen III was calculated from peak sizes using the formula:

Percentage type III collagen = Area α1(III)×1.12×100/[area α1(III)×1.12]+area α1[I] [14], where a calibration factor of 1.12 was used to correct for the color yield from equal weights of the two collagen types [14].

The insoluble precipitate from the papain-digested and centrifuged sample as described above, comprising insoluble collagen and insoluble elastic tissue associated proteins (ETAP) was used for indirect ETAP analysis. The residual insoluble tissue extract was rinsed in PBS three times and distilled water once before freezing and lyophilization for 4 hours. After weighing (W_res_), the residual tissue was sent to Australian Proteome Analysis Facility (APAF, Macquarie University, NSW, Australia) for amino acid analysis. The weight of insoluble collagen in the residual tissue (W_res-col_) was calculated based on its corresponding Hyp amino acid amount. The percentage of ETAP in the original tissue samples was calculated using the following formula: ETAP % = [(W_res_−W_res-col_)/W_dry_]×100. Elastin and ETAP distribution in tissues were also visualized with a histological stain (Verhoff Van Gieson).

Glycosaminoglycan (GAG) content was determined using the colorimetric dimethylmethylene blue (DMMB) assay. Sample preparation was the same as for collagen measurement. After centrifuging, the supernatant was combined with DMMB reagent solution [40 mM NaCl, 40 mM glycine, 0.1 M HCl, 46 mM DMMB (Sigma), pH 3.0] for 2 mins [15]. The concentration of GAG in papain digests was determined spectrophotometrically at 525 nm. Chondroitin sulfate C from shark cartilage (Sigma) was used as the standard (0–0.5 mg/mL).

Vaginal tissue for mechanical testing was dissected from virgin (n = 3), parous (n = 7), and pregnant (n = 6) sheep, and stored at −20°C until testing. Frozen tissues were thawed overnight at 4°C and tested within 24 hours of thawing. Testing of frozen-thawed vaginal tissue does not alter the mechanical properties and is more reliable as the same conditions are used for each specimen [16]. Dog-bone shaped samples of central dimensions 4×34 mm were punched from the sheep anterior and posterior tissues in the longitudinal axis (Fig. 1) and kept moist using PBS. Prior to testing, sample thickness (n = 3) was measured using digital calipers and used to calculate the initial cross sectional area of the sample. Uniaxial tensiometry was performed using an Instron® Tensile Tester (5567®Instron Corp, USA) and a 5 kN load cell. To avoid tissue slippage, samples were secured in pneumatic serrated jaws, which were set to a gauge length of 14 mm. Samples were then preloaded at 10 mm/min to 100 mN and then cyclically loaded from 0 to 1 N, 0 to 2 N, and 0 to 3 N, for 5 cycles each, at 20 mm/min and finally extended to break. This test method is based on the assessment of human vaginal tissue [16]–[19], with loading values chosen to avoid damage to the ovine tissues.

**Figure 2:**
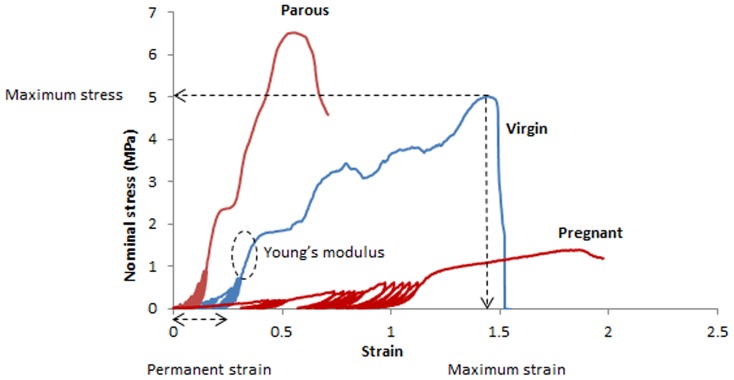
Typical stress-strain curves for parous, virgin and pregnant ovine vaginal tissue, indicating maximum stress and strain, Young's modulus, and permanent strain for the virgin tissue curve.

Stress-strain curves were plotted from the force and elongation data generated, calculating nominal stress (MPa, where 1Pa = 1N/mm^2^) by dividing the force (N) by the initial cross sectional area (mm^2^) and the strain by dividing the extension (mm) by the initial gauge length (mm). The Young's modulus (MPa) was determined from the slope of the stress-strain curve in the linear region [20], [21]. This was identified as the region immediately following cyclic loading and prior to yielding (Fig. 2). Permanent strain (%) was calculated as the percentage increase in sample length following cyclic loading [20]. Maximum stress (MPa) was derived from the stress-strain curve and maximum strain (%) was calculated from the corresponding maximum strain, derived from the stress-strain curve (Fig. 2).

GraphPad Prism 6 was used for statistical analysis. Results are reported as mean ± SEM for each of the three experimental groups. Since the data was not normally distributed (D'Agostino & Pearson omnibus normality test), non-parametric analysis using Kruskal – Wallis ANOVA and post hoc test for comparisons between the different groups (Tukey's correction). Differences between the anterior and posterior wall was determined by paired t-tests. P values<0.05 were considered statistically significant.

## Results

Virgin sheep were one year old, while parous and pregnant sheep were 4 to 5 years old and had delivered 3 lambs prior to the study. The mean gestational age of the fetus in the pregnant sheep was 132±8 days. We developed a clinical score for vaginal wall distensibility by measuring the maximum displacement under traction as shown in Table 1. There was hardly any displacement of the anterior and posterior walls at points 3 cm above the urethral orifice in virgin and parous sheep. In pregnancy, the mean displacement was 3.3 cm with traction of both vaginal walls. There was no difference in displacement of the cervix with traction in any of the sheep.

**Table 1:** Measurements of vaginal wall and cervical maximum displacement under traction.

	Virgin	Parous	Pregnant	p- value
Ba	0.8±0.3	1±0.4	3.3±1	<0.05*
Bp	0.8±0.3	1±0.4	3.2±0.8	<0.05*
C	1.5±0.9	1.5±0.6	1.6±0.8	ns

Data are reported as mean ± SEM in cm for n = 3 virgin and n = 6 each for parous and pregnant ewes.

*compared between virgin versus pregnant and parous versus pregnant.

Ba, reference point 3 cm proximal of the external urethral meatus of the anterior wall. Bp, reference point on the posterior wall opposite Ba. C, Cervix. Ns, not significant.

**Figure 3:**
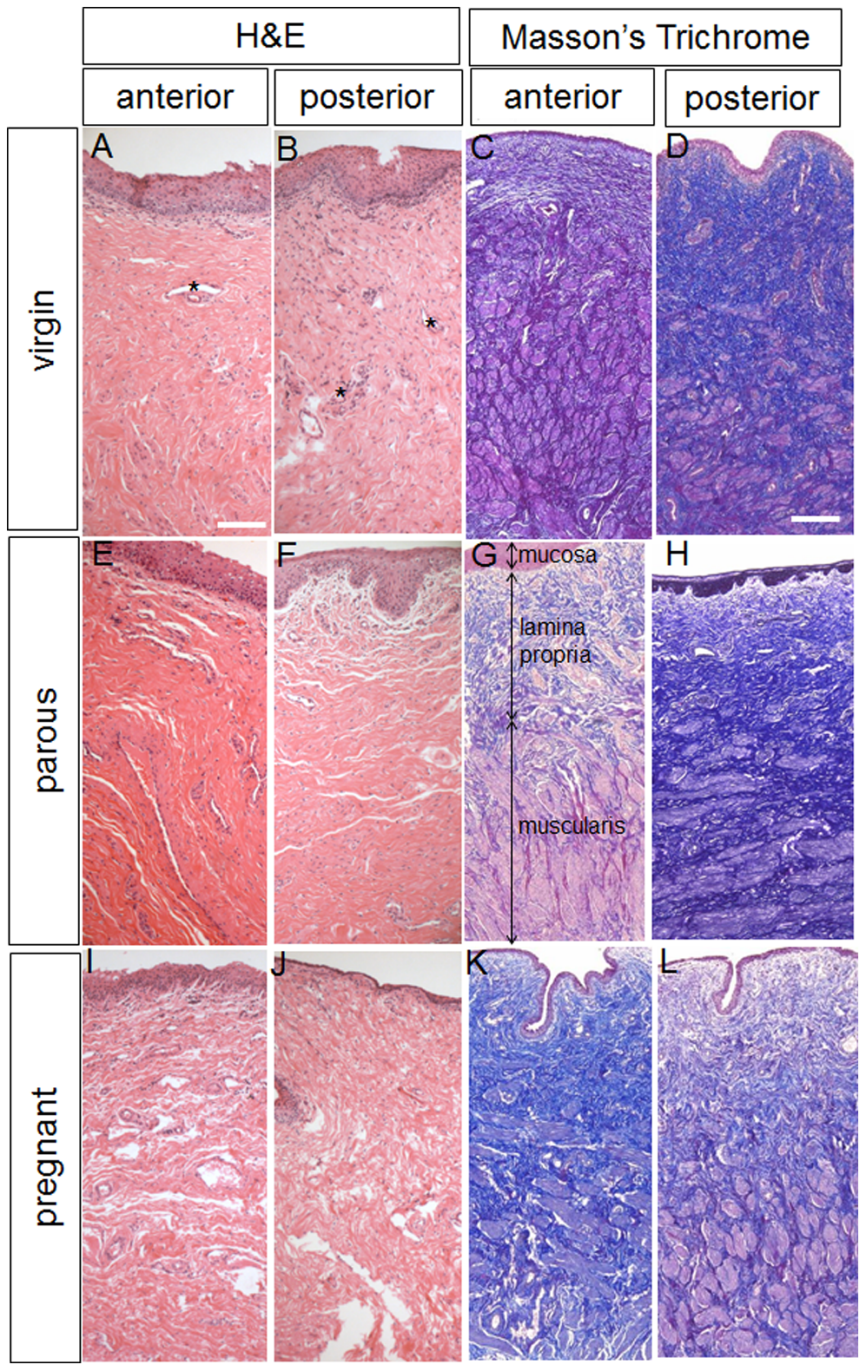
Histological structure of ovine vaginal wall. H+E and Masson stained sections showing the anterior and posterior vaginal walls of virgin (A–D), parous (E–H) and pregnant sheep (I–L). * indicates blood vessels. In image E arrows indicate the 3 vaginal layers. Scale bar for H+E sections 100 µm, for Masson sections 250 µm.

Histologically, the vaginal wall from all groups showed the typical 4 zones from the superficial stratified squamous epithelium, dense connective tissue lamina propria, a muscularis of smooth muscle cells and connective tissue, to the loose connective adventitia (Fig. 3). The virgin sheep (Fig. 3A, B) had a dense connective tissue with moderate uniform cell infiltrates and blood vessels in the lamina propria with densely packed collagen (Fig. 3 C, D). The vaginal architecture of the parous sheep was similar to that of virgin sheep with respect to epithelial height, vessel density (Fig. 3 E, F), and densely packed collagen (Fig. 3 G, H). Pregnant ovine vaginal tissue was strikingly different with fewer cells but similar density of blood vessels in the lamina propria (Fig. 3 I, J) and markedly less dense tissue and collagen packing in all layers (Fig. 3 K,L).

**Figure 4:**
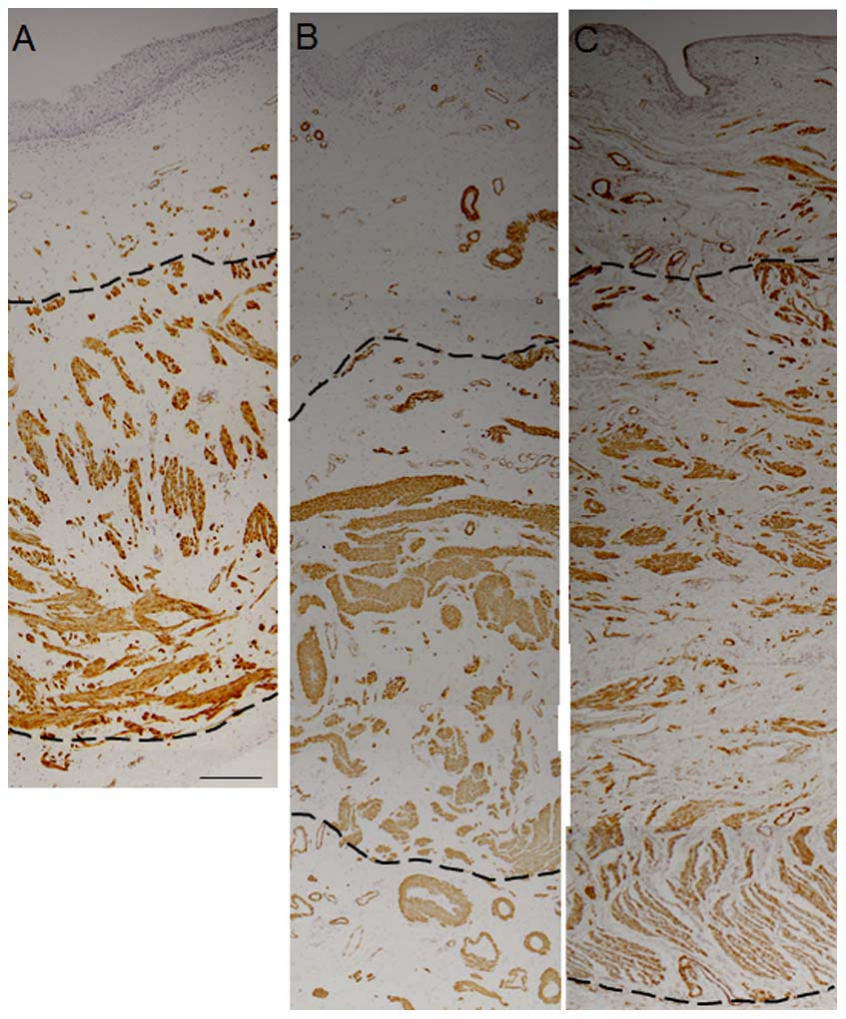
αSMA immunohistochemical staining of ovine vaginal wall. A. virgin, B. parous, C. pregnant ovine vaginal tissue. Dotted lines indicate the muscularis layer. Scale bar 200 µm.

The relative amount of smooth muscle cells (SMC) was quantified by measurement of the extent of the muscularis layer from Masson Trichrome-stained sections of each anterior and posterior vaginal tissue from each group. Alpha smooth muscle actin (αSMA) staining was used to confirm SMC presence within the muscularis (Fig. 4). There were extensive variations in the absolute thickness of the muscularis within and between each group,, due to inter subject variation. In most sections, the different zones of lamina propria, muscularis and adventitia were not uniform in thickness, so Table 2 shows the mean minimum and maximum thickness (µm) from sections from replicate animals. The pregnant group showed the highest proportion of muscularis in the vaginal wall (71.1% and 76.5%, anterior and posterior, respectively) and was significantly greater (p<0.05) than vaginal tissue from virgin sheep (50.5% and 45.0%, anterior and posterior, respectively). Tissue from parous sheep showed the greatest variation in muscularis thickness between anterior (65.1%) and posterior (50.7%) location, although these were not significant. The muscularis of parous posterior vaginal tissue was also significantly less than the posterior vaginal wall of pregnant animals (p<0.05).

**Table 2:** Measurements of the muscularis thickness in virgin, parous and pregnant sheep.

Tissue		Muscularis thickness, (µm ± SEM)		Total muscularis (% of total wall thickness^1^ ± SEM )
		Min^2^	Max^2^	
Virgin	Anterior	1359±144	1970±149	50.5±6.2*
	Posterior	1037±271	2201±439	45.0±9.3**
Parous	Anterior	2047±356	3325±598	65.1±8.1
	Posterior	1537±311	2747±146	50.7±6.9**
Pregnant	Anterior	1983±377	2440±486	71.1±5.9*
	Posterior	2436±193	3428±596	76.5±5.5**

1 Total thickness was measured from the surface, including the epithelium, to the serosal border.

2 numerous measurements (>20) were taken to establish minimum and maximum values for each slide.

p<0.05 (*pregnant anterior compared to virgin anterior; **pregnant posterior compared to virgin posterior and to parous posterior).

**Figure 5:**
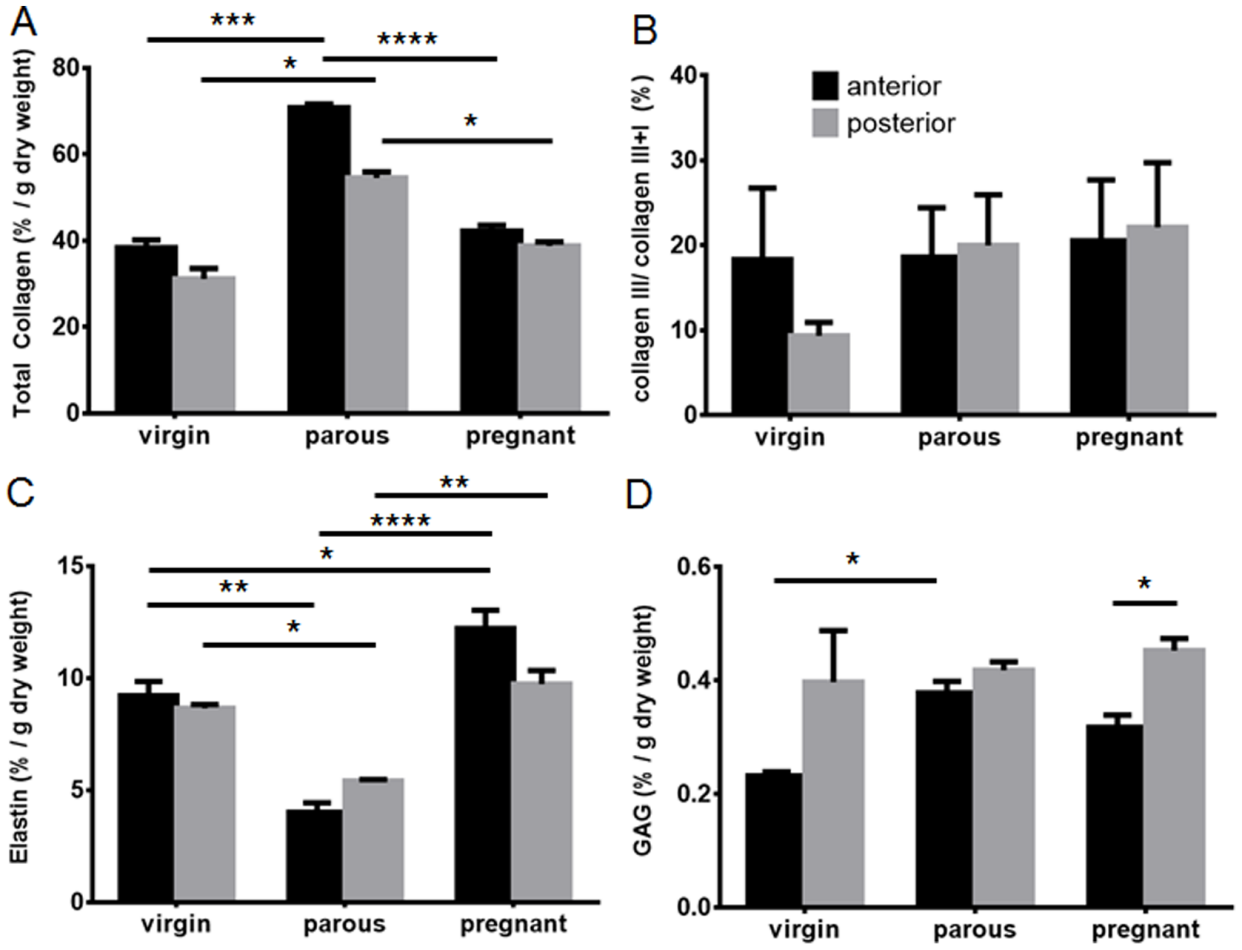
Biochemical analysis of the ECM content of the vaginal wall of virgin, parous and pregnant sheep. A.% total collagen per dry weight assessed by hydroxyproline assay in the anterior (black bars) and posterior (grey bars) vaginal wall. B. % collagen III/(I+III) analyzed by SDS-PAGE. C. % ETAP per dry weight by Amino Acid Analysis. D. % GAG per dry weight by DMMB assay. Data is presented as mean (±SEM), n = 6/group for parous and pregnant and n = 3 for virgin ewes. * p<0.05, *** p<0.001, **** p<0.0001.

The percentage of total collagen relative to tissue dry weight in the anterior wall of parous sheep was significantly higher than in the anterior walls of virgin and pregnant sheep (p<0.001, p<0.0001), respectively (Fig. 5A). Similarly, the posterior vaginal wall from parous sheep had greater total collagen content than tissues from both the virgin and pregnant sheep (p<0.05). The total collagen content was similar between the anterior and posterior vaginal wall for the virgin, parous and pregnant sheep (Fig. 5A). The collagen ratio type III/total collagen type I+III, did not reveal any significant differences between virgin, parous or pregnant groups (Fig. 5B) or between the anterior and posterior vaginal walls (Fig. 5B).

**Figure 6:**
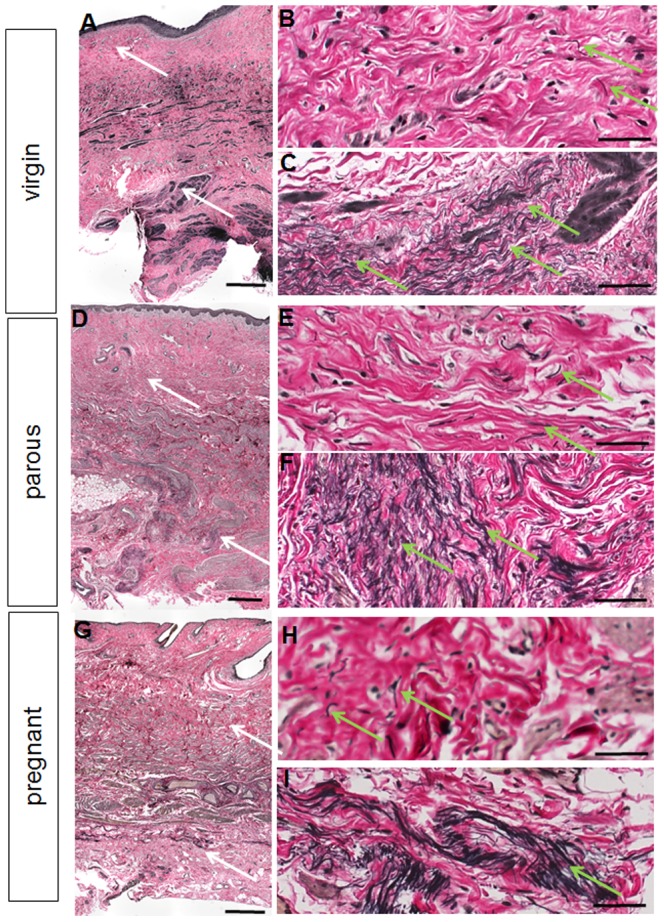
Verhoff's van Gieson staining for elastic fibres (black) on virgin (anterior), parous (posterior) and pregnant (posterior) vaginal tissues. The white arrows on the full thickness images A, D, G denote the region where the higher magnification images were taken in the lamina propria (B, E, H) and deep muscularis (C, F, I). The green arrows indicate regions of elastic fibres. Scale bars 500 µm (full thickness images) and 50 µm (high magnification images).

The ETAP protein content of the parous group was significantly lower in both the anterior and posterior vaginal wall compared to that of the virgin (p<0.01, p<0.05, respectively) and pregnant (p<0.0.0001, p<0.01) ovine tissue (Fig. 5C). The ETAP content of the anterior vaginal wall of the pregnant sheep was significantly higher than the virgin sheep (p<0.05).The anterior and posterior vaginal wall ETAP content was similar within each of the three groups (Fig. 5C). In addition the distribution of elastin and ETAP is demonstrated by a Verhoff Van Gieson histological stain (Fig. 6). The staining points out that elastin (and ETAP) are really a minor component in these tissues with very low levels in the lamina propria and mainly localised in pockets within the deep muscularis.

The total GAG content of virgin, parous and pregnant ovine vaginal tissues was low compared with the other ECM proteins. The virgin sheep showed significantly lower values (p<0.05) in the anterior wall compared to the parous sheep (Fig. 5D). However no differences were observed in the posterior vaginal walls between the groups (Fig. 5D). In contrast to virgin and parous sheep, pregnant sheep showed significantly less GAG in the anterior compared to the posterior vaginal wall. All ECM quantitation were based on %ECM/µg dry weight of tissue. The water content remained similar in all groups (Fig. S1).

**Figure 7:**
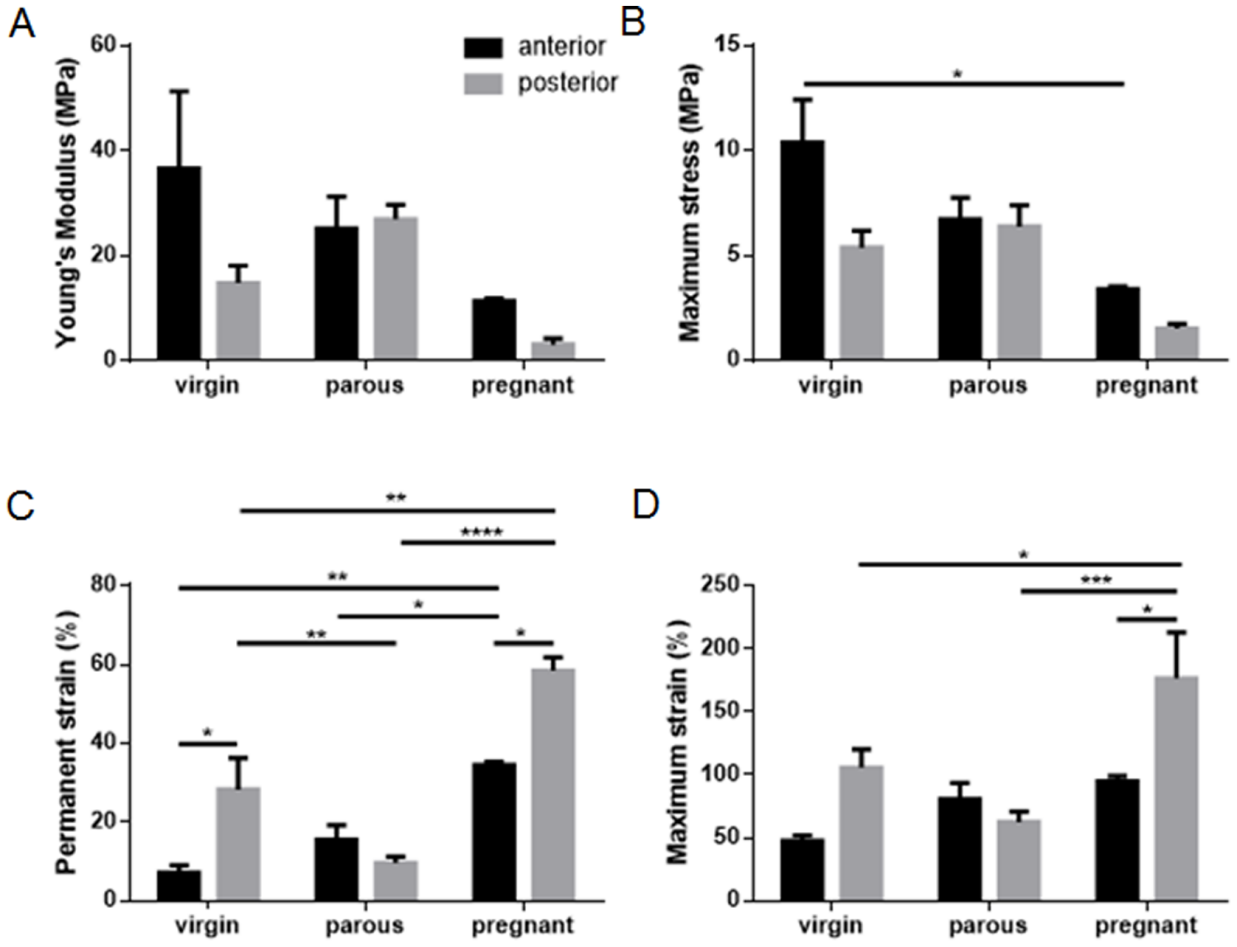
Biomechanical properties of ovine vaginal wall. A. Young's modulus (MPa) B. Maximum stress (MPa). C. Permanent strain (%). D. Maximum strain (%) in virgin, parous and pregnant ewes in the anterior (black bars) and posterior (grey bars) vaginal wall, calculated as described in Figure 2. Data is presented as mean (±SEM), n = 2–7 replicates/group. * p<0.05, **p<0.01, *** p<0.001, **** p<0.0001.

The non-linear stress strain curves showed an immediate region of low stiffness, followed by a region of higher stiffness in the linear region (Fig. 2); cyclic loading occurred in the transitioning region between the low and higher stiffness parts of the curve (Fig. 2). The Young's modulus (Fig. 7A) of the pregnant tissue was smaller than virgin and parous tissue types, but results were not significant. Pregnant tissue also had the smallest maximum stress (Fig. 7B), which was significantly lower than for the anterior tissue of the virgin group (p<0.05). Permanent strain was highest for the pregnant sheep, with significantly higher strains compared to the parous and virgin groups for both anterior (p<0.05, p<0.01) and posterior (p<0.0001, p<0.01) tissues respectively (Fig. 7C). The virgin posterior tissue had significantly higher permanent strain than the parous tissue (p<0.01). Similarly, maximum strain was highest for the posterior tissue of the pregnant group compared to the posterior tissues of the parous (p<0.001) and virgin (p<0.05) groups (Fig. 7D). Significant differences between anterior and posterior tissues were found for permanent strain in virgin and pregnant tissues (both p<0.05), and maximum strain for pregnant tissues (p<0.05), but none were observed for parous tissue for any of the biomechanical parameters examined. Tissues studied exhibited the Mullins effect, showing an increased strain at the same stress level for subsequent loading cycles. Although not quantified, it was observed that this effect was more pronounced for the pregnant tissue than the other tissue types (Fig. 2).

## Discussion

In this study we provide the first comprehensive analysis of normal ovine vaginal tissue composition combining histological, biochemical and biomechanical analyses at different reproductive stages. We also described the first clinical assessment of vaginal wall and cervical displacement with traction. We found that parous sheep had the lowest ETAP and highest collagen content relative to tissue dry weight, and that pregnant sheep had the highest ETAP content and thickest musculature. Pregnant ovine vagina was the most extensible, but weakest tissue, with least dimensional recovery following repetitive loading. The parous and pregnant groups were of equivalent parity, indicating that pregnancy has the greatest influence on ovine vaginal ECM tissue composition and biomechanical properties. In general, there was a trend toward increased stiffness of anterior, compared to posterior tissue, reflecting the findings of other studies, in which anterior POP and non-POP vaginal tissues were stiffer than their posterior tissue counterparts [19]. However, anterior tissue was less extensible and more elastic than posterior vaginal tissue.

A recent review concluded that the clinical data on vaginal tissue composition was inconclusive due to the heterogeneity of reported data and inadequate standardization and quantification of the changes [22]. We showed that the vagina predominantly comprises collagen type I which is largely responsible for its tensile strength. Parous vaginal tissue had the highest total collagen content relative to dry tissue weight which was associated with a high maximum (breaking) stress. In contrast, pregnant tissue had significantly less collagen, which may be associated with the lower maximum stress. We did not find any quantitative differences in the level of collagen type III between pregnant, virgin and parous sheep. Ennen et al examined sheep with antepartum prolapses and found that mRNA expression for collagen type I was decreased, implying that elevated collagen type III may be associated with POP [23].

Elastin is the other major fibrillar protein found in soft viscoelastic tissues. The ETAP content was significantly lower in vaginal tissues from parous sheep. Elastic fibres are normally intended to last a lifetime except in reproductive tissue where high degradation and re-synthesis was observed [24]. The muscles and connective tissue of the vaginal wall are responsible for its mechanical integrity and functionality. Both the lamina propria and the muscularis contribute to the strength and visco-elasticity of the vagina. The changes in the vaginal connective tissue ETAP composition were consistent with our mechanical assessment of these tissues. Pregnant vaginal tissue contained the highest ETAP and thickest muscularis and associated smooth muscle cell content; it was the least stiff (Young's modulus) and most extensible tissue (maximum strain), with an average four-fold decrease in stiffness and two-fold increase in maximum strain, compared to parous sheep of similar parity, and to virgin vaginal tissue. These findings are in agreement with studies in mice [25] and rats [26] where the supportive perivaginal tissues decreased in stiffness and strength during pregnancy and returned to virgin levels four weeks after delivery [26]. However, another rat study showed increased vaginal distensibility during pregnancy compared to virgins, which did not return to virgin levels four weeks postpartum [27]. We also found a higher level of induced permanent strain following cyclic loading in pregnant vaginal tissue, indicating it was the least elastic by this measure and the least likely to return to its original dimensions. Previous studies characterizing hysteresis by cyclic loading found vaginal tissue visco-hyperelastic [24] and to exhibit the Mullins effect [28]; with a smaller stress required to reach a prescribed strain in subsequent loading cycles. In our study, the Mullins effect was more pronounced for pregnant vaginal tissue compared to that from virgin and parous ewes. We suggest that such a large elastic deformation permits the passage of the fetus through the vagina during delivery as described in other studies.

An ideal animal model for prolapse research has not yet been established. Rats and rabbits are cost effective and available in large numbers [24], however, their reproductive tract is small and the pelvic musculature is different from humans [24]. The reproductive tract of sheep is large with similar vaginal dimensions as humans, and with similar pelvic organ support by the three primary levels although the pelvic musculature differs [24]. Furthermore, sheep experience high internal pressure on the pelvic structures compared to other quadruped species due to their ruminant anatomy [8]. The ovine model may therefore be a more suitable preclinical model [24], [29] for evaluating regenerative medicine approaches for transvaginal surgical treatment of POP. Macaques would be an ideal model, but their prohibitive costs and ethical restrictions are much greater than for sheep [24].

In conclusion, our data showed that vaginal tissue is dynamic, undergoing profound changes in connective tissue composition that influence the biomechanical behavior, particularly during pregnancy. We speculate that varying degrees of vaginal tissue recovery might explain why some females develop POP and others do not.

## Supporting Information

Figure S1 **Wet weight of virgin, parous and pregnant sheep, anterior (black bars) and posterior (grey bars) vaginal wall.** Data is presented as mean (±SEM), n = 6/group for parous and pregnant and n = 3 for virgin ewes.
(TIF)
