# Supplementary figures and images for: Influence of Reproductive Status on Tissue Composition and Biomechanical Properties of Ovine Vagina
Source: PLoS One. 2014 Apr 7;9(4):e93172. doi: 10.1371/journal.pone.0093172 (PMC3977844; doi:10.1371/journal.pone.0093172)

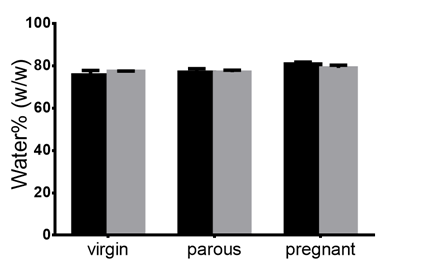

Supplement: Figure S1 — Wet weight of virgin, parous and pregnant sheep, anterior (black bars) and posterior (grey bars) vaginal wall. Data is presented as mean (±SEM), n = 6/group for parous and pregnant and n = 3 for virgin ewes. (TIF) [file pone.0093172.s001.tif]
